# Reconstitution of the embryonic kidney identifies a donor cell contribution to the renal vasculature upon transplantation

**DOI:** 10.1038/s41598-018-37793-z

**Published:** 2019-02-04

**Authors:** Yoichi Murakami, Hidekazu Naganuma, Shunsuke Tanigawa, Toshihiko Fujimori, Masatoshi Eto, Ryuichi Nishinakamura

**Affiliations:** 1 Department of Kidney Development, Institute of Molecular Embryology and Genetics, Kumamoto University, Kumamoto, 860-0811 Japan; 2 Department of Urology, Graduate School of Medical Sciences, Kyushu University, Fukuoka, 812-8582 Japan; 3 Division of Embryology, National Institute for Basic Biology, Aichi, 444-8787 Japan

## Abstract

The kidney possesses a highly organised vasculature that is required for its filtration function. While recent advances in stem cell biology have enabled the *in vitro* generation of kidney tissues, at least partially, recapitulation of the complicated vascular architecture remains a huge challenge. Herein we develop a method to reconstitute both the kidney and its vascular architecture *in vitro*, using dissociated and sorted mouse embryonic kidney cells. Upon transplantation, arteriolar networks were re-established that ran through the interstitial space between branching ureteric buds and eventually entered glomeruli. Using this system, we found that donor-derived endothelial cells significantly contributed to the arterioles and glomerular capillaries formed after transplantation. Unexpectedly, the near-complete depletion of canonical endothelial cells from the donor embryonic kidney suggested the existence of unidentified donor-derived endothelial precursors that were negative for canonical endothelial markers, but still contributed significantly to the vasculature in the transplants. Thus, our protocol will serve as a useful platform for identification of renal endothelial precursors and induction of these precursors from pluripotent stem cells.

## Introduction

The kidney possesses a highly organised vasculature that is required for its filtration function. In recent years, several groups, including ours, have succeeded in inducing kidney organoids from mouse embryonic stem cells (ESCs) and/or human induced pluripotent stem cells (iPSCs)^1–3^. However, recapitulation of the complicated renal vascular architecture remains a huge challenge. While attempts to mix organoids and endothelial cells (ECs) were reported in other organs^4,5^, vascularisation of kidney organoids has additional hurdles to overcome. The kidney receives abundant blood flow (20–25% of cardiac output) through the renal arteries and filters the serum component to produce urine in the glomeruli. The urine flows through the renal tubules and collecting ducts, and eventually exits the kidney via the ureter, while the blood flow passes through the glomeruli, distributes throughout the kidney, and finally leaves the kidney through the renal veins^6,7^. Thus, reproduction of the organised architectures in both the kidney and the vasculature is essential for functional kidney reconstruction, and generation of the renal arteriolar network that reaches the glomeruli is the initial step in achieving this goal.

Most reports utilising human iPSCs have described the induction of nephron progenitors (NPs) that give rise to glomeruli and renal tubules^1–3^. However, the majority of the glomeruli in the *in vitro* organoids remained avascular, although CD31+ ECs were detected in the interstitial space in close proximity to the glomeruli^2,8^ (and our unpublished observations). In contrast, we previously reported that the glomeruli were heavily vascularised when NPs induced from human iPSCs were transplanted beneath the kidney capsule of immunodeficient mice^8^, although a prominent renal artery-like vasculature was not formed. Interestingly, the ECs in the glomeruli were mostly derived from the host mice. This finding is reminiscent of reports on interspecies kidney transplantation, including chick, quail, and mouse, that claimed a host-derived origin of the renal glomerular vasculature^9,10^. Meanwhile, other reports utilising embryonic kidneys from genetically labelled mice showed a contribution of donor-derived ECs to the transplanted tissues^11–13^. Furthermore, a recent report on transplantation of human iPSC-derived kidney organoids revealed the existence of human ECs in the glomeruli to some extent^14^. Therefore, it is critical to determine the donor versus host contribution to renal ECs after transplantation, especially in the kidney organoid setting, for recapitulation of the renal vasculature. In addition, kidney organoids would be beneficial for detection of renal EC precursors and their development, because candidate cell fractions can be sorted and reaggregated into organoids.

While reaggregation of the embryonic kidney from a single-cell suspension was reported^15,16^, few have succeeded in reconstituting the arteriolar structure and glomerular vasculature. We recently reported the reconstitution of a higher-order kidney structure from mouse ESCs and dissociated embryonic kidneys^17^. Our method utilised three progenitors of the kidney: Itga8+ NPs that give rise to glomeruli and renal tubules, Pdgfra+ stromal progenitors (SPs) that form interstitial cells, and the ureteric bud (UB) that gives rise to collecting ducts and ureters. When NPs and UBs (isolated from embryonic kidneys or induced from mouse ESCs) and embryonic kidney-derived SPs were aggregated, three-dimensional kidney-like structures were generated *in vitro*, consisting of branching UB trees with nephrons (glomeruli and renal tubules) distributed at the UB tips^17^. We reasoned that incorporation of ECs into this system would facilitate the generation of vascularised organoids, and also elucidate the donor versus host origin of ECs after transplantation. In this study, we successfully generated vascularised kidney organoids equipped with branching UBs and nephrons. Upon transplantation, intrarenal arteriolar structures were formed in the space between the branching UBs, and the glomeruli were vascularised. Furthermore, we unexpectedly identified the existence of EC precursors that can dominantly contribute to the vasculature upon organoid transplantation.

## Results

### Extrarenal and intrarenal vasculature framework is established by E15.5

**Figure 1:**
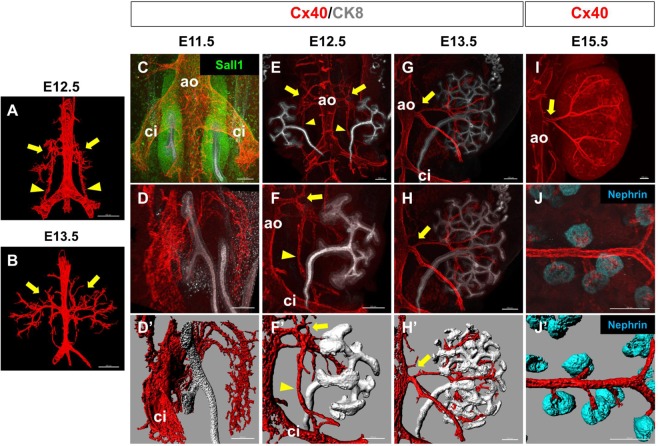
Extrarenal and intrarenal vasculature framework is established by E15.5 (**A**,**B**) Arteries at E12.5 (**A**) and E13.5 (**B**) reconstituted from serial sections immunostained for CD31. Arrows: renal arteries budding from the aorta (ao); arrowheads: collateral arteries connecting the renal arteries and the common iliac artery . (**C**,**D**) Whole-mount staining of arteries (Cx40) and the kidney (NP: Sall1 and/or UB: CK8) at E11.5. (**C**) Dorsal view. The kidney is located on the ventral side of the common iliac arteries (ci). (**D**) Right oblique view of the right kidney. (D’) Digitised image of (**D**). No apparent arterial connection to the kidney was observed. ao: aorta. (**E**–**H**) Whole-mount staining of arteries (Cx40) and the kidney (UB: CK8) at E12.5 (**E**,**F**) and E13.5 (**G**,**H**). Dorsal views of the right kidneys are shown. (F’,H’) Digitised images of (**F**,**H**), respectively. Arrows: renal arteries budding from the aorta (ao) and invading into the kidneys; arrowheads: collateral arteries connecting the renal arteries and the common iliac artery (ci). At E13.5, the collateral arteries had disappeared. (**I**) Whole-mount staining of arteries (Cx40) at E15.5. A dorsal view of the right kidney is shown. Arrow: a single stalk for the renal artery is formed. (**J**) Whole-mount staining of intrarenal arterioles (Cx40) connected to glomeruli (Nephrin). (J’) Digitised image of (**J**). Three mice were examined at each stage and representative images are shown. Scale bars: 100 µm.

To examine when and how the renal vasculature is formed *in vivo*, we stained serial sections of embryonic day (E) 12.5 and E13.5 mouse embryos for CD31 (Fig. 1A,B). We then reconstructed three-dimensional images by colouring the CD31 + ECs continuous with the aorta in red (putative arteries and arterioles) and those continuous with the vena cava in blue (putative veins) (Supplementary Fig. S1). While well-formed renal arteries were observed at E13.5, those at E12.5 appeared to arise from not only the abdominal aorta, but also the common iliac arteries (Fig. 1A). Veins were formed as plexuses around the arteries, as confirmed by whole-mount staining of a vein marker, Nrp2^18,19^ (Supplementary Fig. S1).

We further performed whole-mount immunofluorescence staining for Connexin 40 (Cx40), an arteriole marker^20,21^, at the initial stages of kidney development. At E11.5, the kidney was located on the ventral side of the common iliac arteries. While small vessels surrounded the kidney, no clear vascular connection to the kidney was observed (Fig. 1C,D, D’). At E12.5, short stalks budded out from the aorta and started to branch into the kidney (Fig. 1E,F, F’). One of the branches elongated caudally and connected to the common iliac artery, consistent with the findings in Fig. 1A. At E13.5, this connection disappeared, but branched arteries invaded deep into the kidney through the space between the UBs (Fig. 1G,H, H’). At E15.5, the stems of the arteries were unified into one renal artery, and many tips branched out from the arterioles and connected to the glomeruli (Fig. 1,J,J’). These results suggested that the initial vascular connection in the kidney occurred at E11.5–E12.5 and the overall framework of the extrarenal and intrarenal vasculature was established by E15.5.

### Endothelial cells invade the kidney at E11.5

**Figure 2:**
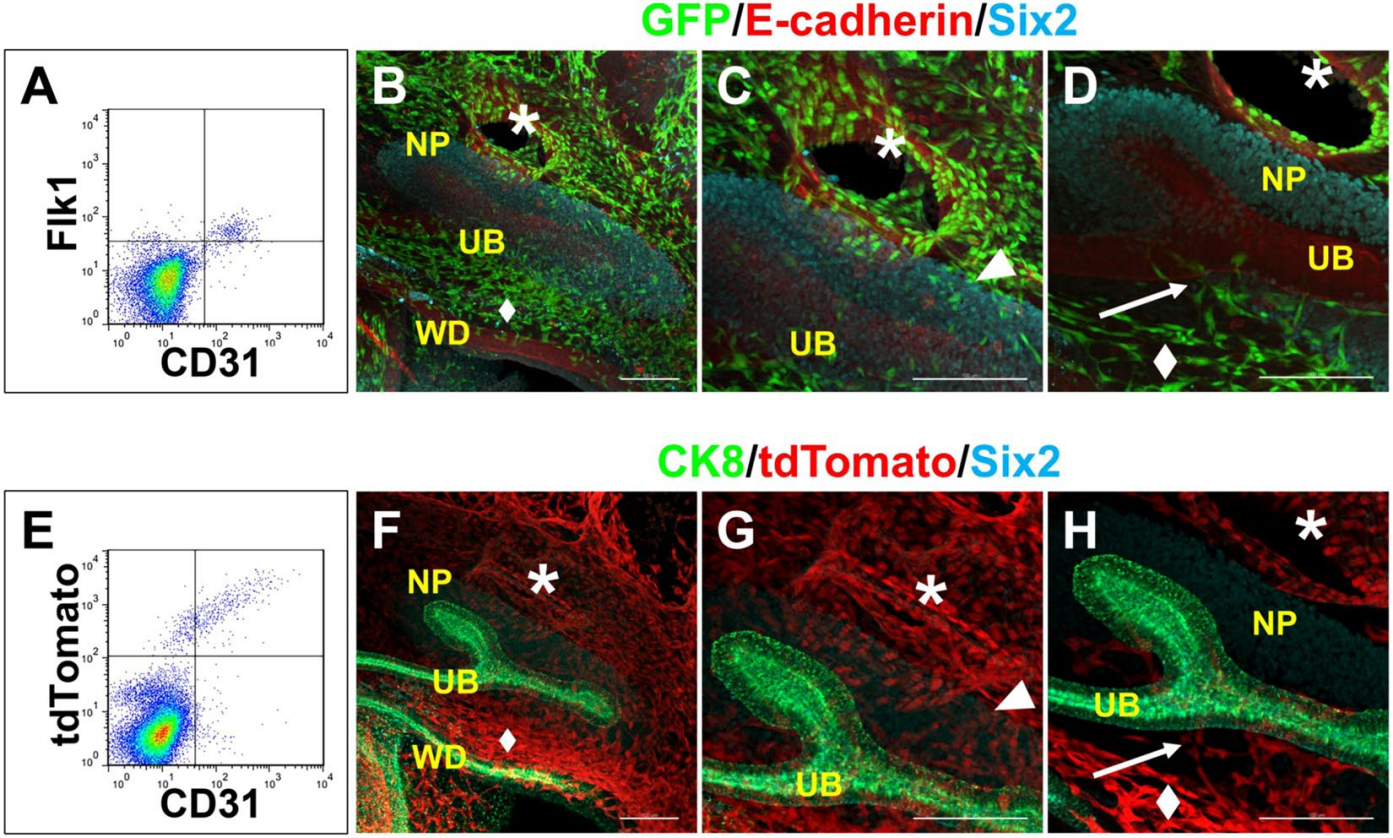
Endothelial cells invade the kidney at E11.5 (**A**) Flow cytometry analysis of E11.5 wild-type kidneys. The majority of the CD31+ cells are Flk1. Representative data from three independent experiments are shown. (**B**–**D**) Whole-mount staining of GFP+ ECs around the E11.5 Flk1-GFP kidney (NP: Six2; UB: E-cadherin). Magnified images of the dorsal (**C**) and ventral (**D**) sides of the kidney at different Z-planes are also shown. (**E**) Flow cytometry analysis of E11.5 Tie2Cre;tdTomato kidneys. The majority of the CD31+ cells are tdTomato+. (**F**–**H**) Whole-mount staining of tdTomato+ ECs around the E11.5 kidney (NP: Six2; UB: CK8). Magnified images of the dorsal (**G**) and ventral (**H**) sides of the kidney at different Z-planes are also shown. Arrows: ECs invading the kidney along the UB stalk, from the plexus (diamonds) along the Wolffian duct (WD); arrowheads: ECs surrounding the NPs; asterisks: common iliac artery. Scale bars: 100 µm. Representative images from three mice for each genotype are shown.

Flow cytometry analysis of the dissociated E11.5 kidneys showed the presence of CD31+ and/or Flk1+ populations (Fig. 2A). Because the majority, if not all, of the CD31+ cells were Flk1+, we utilised Flk1-GFP mice^22^ to analyse the detailed spatial distribution of ECs around the kidney at E11.5. Although no clear arterial connection was observed as described above, numerous Flk1+ ECs surrounded the developing kidney (Fig. 2B). On the dorsal side of the kidney, ECs appeared to be derived from the common iliac arteries and reached, but did not penetrate, the NP region in the kidney (Fig. 2B,C). On the ventral side, ECs were distributed in the space along the Wolffian duct (Fig. 2B), and some of them ran toward the kidney along the UB (Fig. 2D). We also examined Tie2Cre;tdTomato mice^23,24^ at E11.5 and found that the majority of CD31+ cells were tdTomato+ by flow cytometry (Fig. 2E). The spatial distribution of tdTomato+ ECs was similar to that in Flk1-GFP mice (Fig. 2F–H). Thus, ECs invade into the dorsal and ventral sides of the kidney at E11.5. These findings were at least partially consistent with recent reports on kidney vasculature development^25–27^.

### Vascular development is limited in the reconstituted kidney organoids *in vitro*

**Figure 3:**
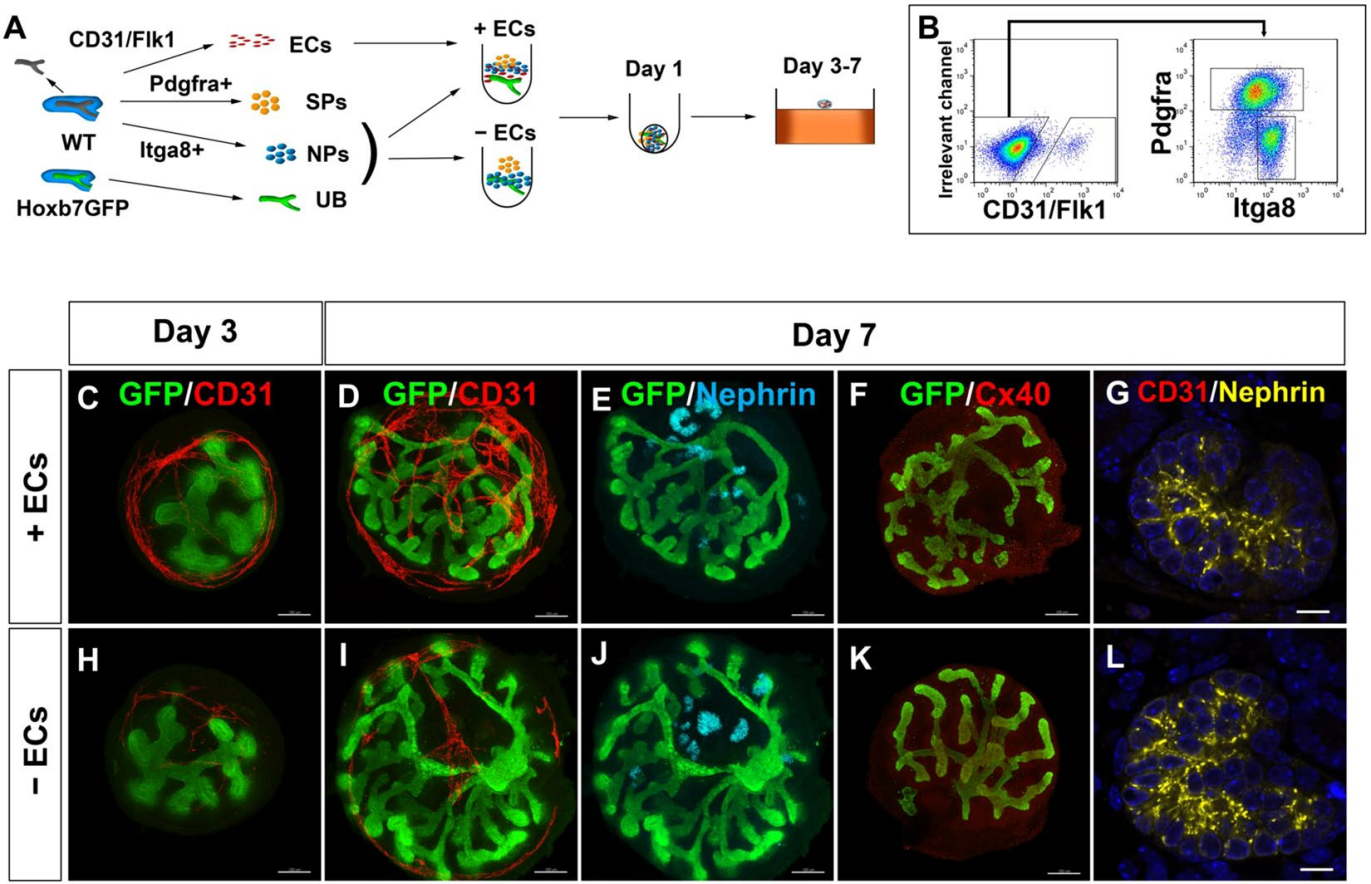
Vascular development is limited in the reconstituted kidney organoids *in vitro* (**A**) Scheme for kidney organoid reconstitution and culture *in vitro*. Three types of kidney progenitors at E11.5 (NPs, SPs, and Hoxb7-GFP+ UBs) were aggregated overnight with or without ECs. The organoids were subsequently cultured at the air-liquid interface for 2–6 days (days 3–7). (**B**) Sorting of ECs, NP, and SPs. CD31+ and/or Flk1+ cells were sorted as ECs (left), and the cells in the CD31−/Flk1− fraction (left) were further sorted into Itga8+ NP and Pdgfra+ SP fractions (right). (**C**–**F**) Whole-mount staining of kidney organoids aggregated with ECs and cultured for the indicated days. GFP+ UB branching and CD31+ vasculature formation (**C**,**D**), as well as Nephrin+ glomerulus formation (**E**), were observed. No Cx40+ arterioles (**F**) were observed. (**G**) Section staining of glomeruli (nephrin). No CD31+ ECs were detected in glomeruli. (**H**–**L**) Whole-mount (**H**–**K**) or section (**L**) staining of kidney organoids aggregated without ECs and cultured for the indicated days. No apparent differences between the organoids with (**C**–**G**) and without (**H**–**L**) ECs were observed. Scale bars: 100 µm (**C**–**F**, **H**–**K**); 10 µm (**G**,**L**). Representative images of three organoids each with and without ECs from three independent experiments are shown.

We recently reported the reconstitution of a higher-order kidney structure from dissociated mouse embryonic kidneys^17^. To examine the vascular development in the reconstituted kidney organoids, we dissociated E11.5 kidneys and combined them with ECs. CD31+ and/or Flk1+ cells were sorted as ECs, and the cells in the CD31−/Flk1− fraction were further sorted into Itga8 + NP and Pdgfra+ SP fractions (Fig. 3A,B). The cells were reaggregated with UBs from Hoxb7-GFP mice^28^, which expressed GFP and enabled monitoring of UB branching. During culture, the UBs branched extensively (Fig. 3C,D) and numerous glomeruli were observed in the periphery at day 7 (Fig. 3E), indicating successful reconstitution of the kidney structure. Whole-mount immunostaining for CD31 showed the formation of a vascular network around the branching UBs (Fig. 3C,D). When ECs were not combined with the other kidney components (NPs, SPs, UB), vascular formation was hampered, while UB and glomerular development was comparable to the findings in the presence of ECs (Fig. 3H–J).

Expression of Cx40 was almost undetectable and much weaker than that in the freshly isolated intact kidney, even when ECs were combined for kidney organoid formation (Fig. 3F,K, Supplementary Fig. S2). Section staining showed the presence of ECs in the interstitial space of the kidney (Supplementary Fig. S2), but the cells failed to integrate into the glomeruli (Fig. 3G,L). These results showed that our reaggregation method could generate vascularised kidney organoids *in vitro*, although arteriolar development and glomerular vascularisation were still limited.

### Transplanted kidney organoids form an arteriolar network and glomerular capillaries

**Figure 4:**
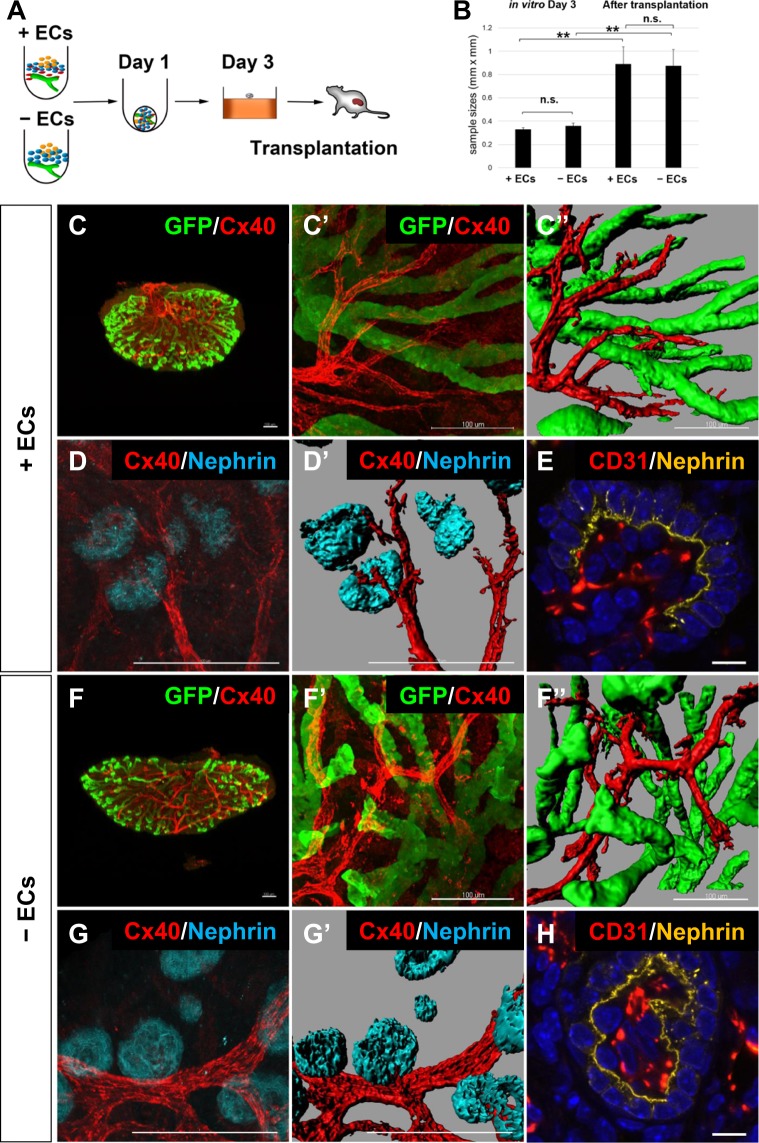
Transplanted kidney organoids form an arteriolar network and vascularised glomeruli (**A**) Scheme for kidney organoid transplantation. The organoids were generated as shown in Fig. 3A, transplanted at day 3, and harvested at 7 days after transplantation. (**B**) Size growth of the organoids from day 3 (before transplantation) to 7 days after transplantation. Size was indicated as short axis (mm) × long axis (mm). **p < 0.01; n.s.: not significant (n = 5). (**C**) Whole-mount staining of organoids with ECs at 7 days after transplantation. (C’) Magnified image of (**C**). (C”) Digitised image of (C’). Cx40+ arterioles were observed to run through the space between the branched UBs. (**D**) Cx40+ ECs were connected to Nephrin+ glomeruli. (D’) Digitised image of (**D**). (**E**) Section staining showing integration of CD31+ ECs into Nephrin+ glomeruli. (**F**–**H**) Whole-mount (**F**,**G**) or section (**H**) staining of organoids without ECs at 7 days after transplantation. No apparent differences between organoids with (**C**–**E**) and without (**F**–**H**) ECs were observed. Representative images of two organoids each with and without ECs from two independent transplantation experiments are shown. Scale bars: 100 µm (**C,D**,**F**,**G**); 10 µm (**E**,**H**).

Because of the limited vascular development in the kidney organoids *in vitro*, we transplanted reconstituted kidney organoids (day 3 of culture) under the renal capsules of immunodeficient mice (Fig. 4A) using our previously reported method^8,29^. When harvested at day 7 after transplantation, the tissues were significantly larger than their size at transplantation (Fig. 4B) and the GFP+ UBs had branched extensively (Fig. 4C). A Cx40+ branching network was observed to run through the space between the UBs (Fig. 4C’,C”) and reach the glomeruli (Fig. 4D,D’), indicating arteriole formation in the kidney organoids. Furthermore, CD31+ ECs were not only detected in the interstitial regions of the tissues, but also integrated into all the NEPHRIN+ glomeruli examined (n = 19; Fig. 4E), in sharp contrast to the situation *in vitro*. Thus, reconstitution of embryonic kidneys and subsequent transplantation successfully restored the arteriolar network development and glomerular vascularisation, although the running patterns of the arterioles did not completely recapitulate those *in vivo*.

To examine the necessity for ECs in the transplantation assay, we removed CD31+and/or Flk1+ECs from the dissociated metanephric mesenchyme and performed the same reconstitution and transplantation procedures (Fig. 4A). Unexpectedly, the tissue growth (Fig. 4B), UB branching, and arteriolar network formation were comparable to those with ECs (Fig. 4F,F’,F”) and the glomeruli were vascularised with ECs (n = 25; Fig. 4G,G’,H). These results raised two possibilities: the host ECs compensate for the absence of donor ECs, or some EC precursors still exist in the donor tissues.

### Donor-derived ECs contribute to the majority of the renal vasculature upon transplantation

**Figure 5:**
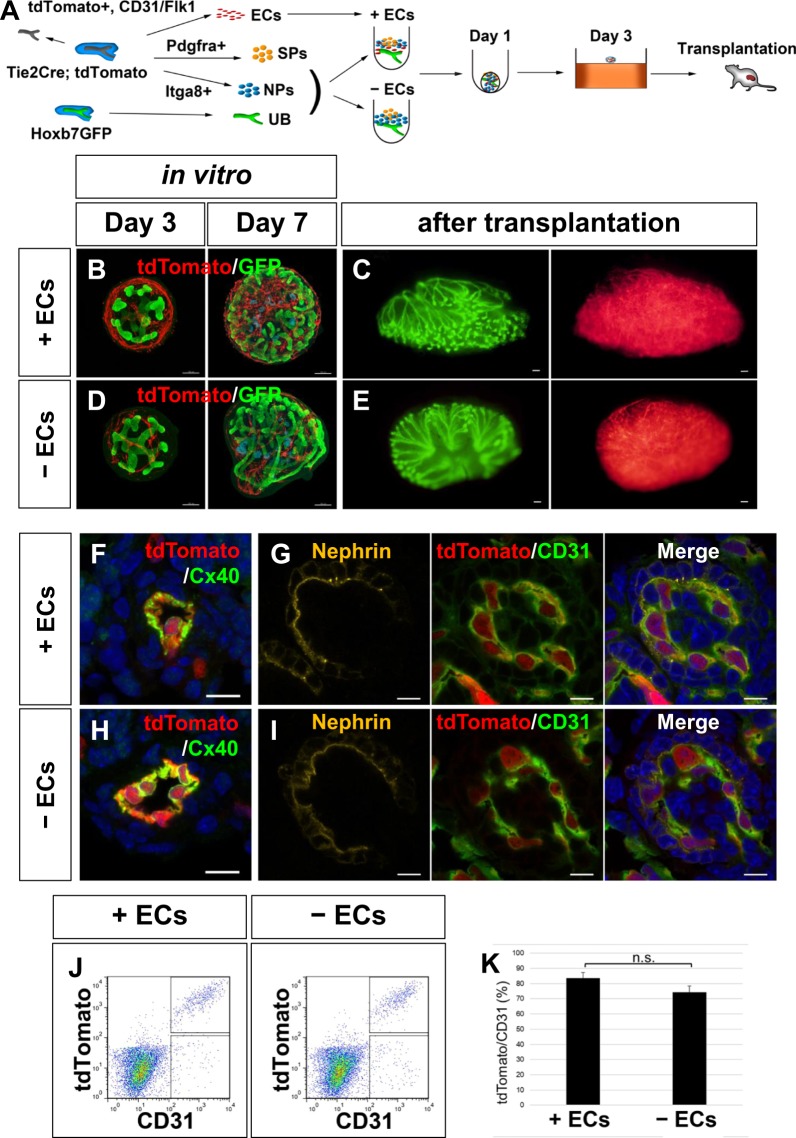
Donor-derived ECs contribute to the majority of the renal vasculature upon transplantation (**A**) Scheme for kidney organoid transplantation utilising Tie2Cre;tdTomato kidneys. NPs and SPs from Tie2Cre;tdTomato E11.5 kidneys were aggregated with Hoxb7-GFP UBs with or without ECs from Tie2Cre;tdTomato kidneys. (**B**) Organoids with ECs cultured for 3–7 days *in vitro*. A tdTomato+ donor-derived vasculature was formed. (**C**) Organoids with ECs at 7 days after transplantation. Branched UBs and extensive distribution of tdTomato+ ECs were observed. (**D**) Organoids without ECs cultured for 3–7 days *in vitro*. The tdTomato+ vasculature formation was limited. (**E**) Organoids without ECs at 7 days after transplantation. An extensive distribution of tdTomato+ ECs was observed. (**F**–**I**) Section staining showing the contribution of tdTomato+ donor-derived cells to the arterioles and glomerular capillaries at 7 days after transplantation. (**F**,**G**) with ECs; (**H**,**I**) without ECs. (**J**,**K**) Flow cytometry analysis of CD31 versus tdTomato in organoids at 7 days after transplantation. The percentages of tdTomato+ donor cells among CD31+ ECs are shown in (**K**). No significant differences were observed between organoids with and without EC. n.s.: not significant (p = 0.14; n = 5). Representative images of five organoids each with and without ECs from three independent transplantation experiments are shown. Scale bars: 100 µm (**B**–**E**); 10 µm (**F**–**I**).

To examine the possibilities described above, we utilised Tie2Cre;tdTomato mice^23^, which express tdTomato in ECs. Initially, we transplanted intact E11.5 kidneys from Tie2Cre;tdTomato mice, and found that the donor cells contributed the majority of the vasculature in the grafted tissues (Supplementary Fig. S3), consistent with previous results^11–13^. We then sorted Itga8+ NPs and Pdgfra+ SPs from the CD31−/Flk1−/tdTomato− fraction and CD31+ and/or Flk1+ /tdTomato+ ECs from Tie2Cre;tdTomato mice, and aggregated them with Hoxb7-GFP+ UBs to reconstitute kidney organoids (Fig. 5A, Supplementary Fig. S3). When cultured for 7 days *in vitro*, tdTomato+ vascular structures were formed in the organoids (Fig. 5B), consistent with the results shown in Fig. 3. Upon transplantation (day 7), tdTomato signals were detected in the entire organoids (Fig. 5C). When exogenous ECs were not combined for organoid generation, formation of a tdTomato+ vasculature *in vitro* was modestly reduced (p < 0.05; Fig. 5D, Supplementary Fig. S2). However, upon transplantation, tdTomato signals were unexpectedly detected in the entire organoids (Fig. 5E). Section staining showed that tdTomato+ cells contributed to the Cx40+ arterioles and glomerular capillaries (Fig. 5F,G), irrespective of the presence of exogenous ECs in the transplanted organoids (Fig. 5H,I). Flow cytometry analysis further revealed that tdTomato+ cells constituted 75–85% of the CD31+ ECs in both cases (Fig. 5J,K), although the presence of exogenous ECs produced slightly higher percentages (83.6 ± 3.8% with ECs; 74.4 ± 4.2% without ECs). These data suggest that the CD31−/Flk1−/Tie2− fraction in the E11.5 kidney may contain EC precursors that can contribute more efficiently to the vasculature of the transplanted organoids than the host ECs. Finally, we combined CD31+ and/or Flk1+ /tdTomato+ ECs from E11.5 Tie2Cre;tdTomato mice with NPs and SPs from the CD31−/Flk1− fraction, as well as UBs, from wild-type mice. Upon transplantation, only 4.57 ± 0.88% of CD31 + ECs in the organoids were positive for tdTomato (n = 7 from 3 independent transplantation experiments). Because the majority of the vasculature in the transplanted organoids was derived from the donor, as shown in Fig. 5J,K, endothelial recruitment from the CD31−/Flk1− fraction is likely to occur even in the presence of exogenous ECs.

## Discussion

Herein, we have established *in vitro* and *in vivo* protocols that can enable vascular development in kidney organoids. Because we identified that the vasculature started to invade the kidney at E11.5, we isolated four populations, NPs, SPs, UB, and ECs, from this stage of kidney development and reconstituted the kidney structures. The kidney organoids *in vitro* exhibited vascular development along with extensive UB branching and glomerulus formation. Transplantation of the organoids further conferred arteriolar network formation, as well as glomerular vascularisation. By using these assays, we revealed the presence of unidentified EC precursors in the donor embryonic kidney that can efficiently contribute to the vasculature upon transplantation.

Our data are consistent with previous reports showing the dominance of donor-derived ECs after transplantation of genetically labelled intact embryonic kidneys^11–13^. However, one of the main differences is that ECs, as well as NPs and SPs, were completely dissociated in our study. Even in this situation, reaggregated embryonic donor cells could reorganise the arteriolar architecture and dominate over the infiltrating host adult ECs. These findings suggest a potential advantage of embryonic ECs over adult ECs for renal vasculature reorganisation upon transplantation.

Unexpectedly, we found that the CD31−/Flk1−/Tie2− fraction in the embryonic kidney contains EC precursors that contributed to the majority of the vasculature upon transplantation. Although the cell types comprising these EC precursors with such robust potential remain unknown, our data are at least partially consistent with a previous report showing the existence of CD146+/CD31− EC precursors that can contribute to the vasculature during *in vitro* culture^30^. Thus, it would be worthwhile to test the CD146+ fraction in our transplantation assays. Once the fraction containing the EC precursors is identified, it could be an ideal target for induction from ESCs/iPSCs for the generation of vascularised kidney organoids. While CD31+ EC-like cells were detected in human iPSC-derived kidney organoids^2^, their inefficient contribution to the vasculature upon transplantation^8^ may suggest an incomplete quality of the induced CD31+ ECs or an absence of the CD31− EC precursors. The ECs in the organoids may be derived from the intermediate mesoderm, because they are induced simultaneously with the kidney^2^. However, the precise origin and developmental pathway of physiological renal ECs remain largely unknown. Elucidation of these issues, combined with the present assay, will facilitate the induction of fully competent renal ECs from ESCs/iPSCs.

One of the advantages of our protocol is that arteriolar network formation can be observed along with extensive UB branching, the latter of which is lacking in most other published methods. Furthermore, nephrons are formed efficiently both *in vitro* and after transplantation, the latter of which achieved glomerular vascularisation. The failure of arteriolar character acquisition and glomerular vascularisation *in vitro* may arise through suboptimal culture conditions, including culture medium components, oxygen concentrations, and flow-mediated physical force. While new techniques such as organ-on-a-chip may solve these issues in the future^31^, transplantation appears to satisfy most of the necessary conditions. In particular, blood flow, which is required for EC development and maintenance^32–34^, is likely to promote development and re-organisation of the newly synthesised vasculature. We detected anuclear red blood cells around the UBs in the transplanted organoids (Supplementary Fig. S3), suggesting that the vasculature of the organoids is likely to be connected to the host circulation. However, dye injection into the host circulation should be performed for formal proof.

The transplanted organoids still differed from kidneys *in vivo*. Our data showed that the trunk of the extrarenal artery was formed around E13.5 *in vivo*, but was not observed in the transplanted kidney organoids. The branching patterns of intrarenal arterioles also differed from those *in vivo*. In addition, ureters were shortened within 3 days upon aggregation (prior to transplantation), and re-elongation of ureters was not observed upon transplantation (see Hoxb7-GFP UBs in Fig. 3–5). These may partly arise because we simply reaggregated NPs and SPs as bulk populations that could have lost their topological information. In particular, SPs consist of multiple populations located in distinct regions^35^, and preservation of this positional information in organoids would be necessary for recapitulation of the complex renal vascular development, as well as formation of a single elongated ureter. Ultimately, the existence of flow in the vasculature and urine production should be examined to prove the functions of the reconstituted kidneys.

In conclusion, our protocol, which can reconstruct the overall kidney and vascular structures from dissociated and sorted cells, will serve as a useful basis for the identification of renal EC precursors and induction of such precursors from ESC/iPSCs, which will eventually lead to the generation of vascularised and functional kidney organoids.

## Methods

### Animals

Flk-1 GFP mice (017006)^22^, Hoxb7-GFP mice (016251)^28^, and R26R-tdTomato mice (007905)^24^ were purchased from Jackson Laboratory. Tie2Cre mice^23^ were kindly provided by Dr. Masashi Yanagisawa (Tsukuba University and The University of Texas Southwestern Medical Center). The primers used for genotyping were: Cre1 (5′-AGGTTCGTTCACTCATGGA-3′) and Cre2 (5′-TCGACCAGTTTAGTTACCC-3′) for the Cre allele (250-bp product); EGFP-F1 (5′-TGAACCGCATCGAGCTGAAGGG-3′) and EGFP-R1 (5′-TCCAGCAGGACCATGTGATCGC-3′) for the eGFP allele (300-bp product); RosaTomato-1 (5′-AAGGGAGCTGCAGTGGAGTA-3′), RosaTomato-2 (5′-CCGAAAATCTGTGGGAAGTC-3′), RosaTomato-3 (5′-GGCATTAAAGCAGCGTATCC-3′), and RosaTomato-4 (5′-CTGTTCCTGTACGGCATGG-3′) for the mutant allele (196-bp product) and wild-type allele (297-bp product). For the transplantation experiments, immunodeficient 8–10-week-old male mice (NOD/ShiJic-scid Jcl) were purchased from CLEA Japan Inc. and housed in a specific pathogen-free animal facility. All animal experiments were performed in accordance with our institutional guidelines and approved by the licensing committee of Kumamoto University (A29–040).

### Organoid reconstitution and culture *in vitro*

The dissociation and reaggregation methods were described previously^17^. Briefly, E11.5 mouse embryonic kidneys were dissociated by sequential incubation in 1 mg/ml collagenase (Sigma; C9407) for 5 min and 0.05% trypsin/EDTA (Thermo Fisher Scientific; 25300054) at 37 °C for 5 min. ECs were sorted as CD31+ and/or Flk1+ fractions by staining the dissociated cells with a combination of anti-CD31 and anti-Flk1 antibodies. The CD31−/Flk1− cells were further divided into Itga8+/Pdgfra− NP and Pdgfra+ SP fractions. After sorting, 25,000 NPs, 50,000 SPs, and 2250 ECs were mixed, seeded into a 96-well low cell-binding U-bottom plate (Thermo Fisher Scientific; 174929), and pelleted by centrifugation (1000 rpm, 4 min). Manually isolated E11.5 UBs from Hoxb7-GFP mouse kidneys were placed onto the deposited sheets of NPs, SPs, and ECs. The kidney reconstitution medium comprised DMEM/F12 (Thermo Fisher Scientific; 11320-033) supplemented with 1% insulin-transferrin-selenium, 0.1 µM dexamethasone, 10 mM nicotinamide, 5 mM HEPES, 5% KnockOut Serum Replacement (Thermo Fisher Scientific), 2 mM L-glutamine, 50 µM 2-mercaptoethanol, and 0.5% penicillin/streptomycin^17^. After 24 h (day 1), aggregated spheroids were transferred onto atelocollagen membranes (Koken; CM-6), with the lower chamber containing a 1:1 mixture of DMEM/F12 (Thermo Fisher Scientific; 11320-033) and EGM-2 (Lonza; CC-3162; without serum) supplemented with 10% foetal bovine serum (FBS), and cultured at the air-liquid interface for a further 2–6 days (days 3–7).

### Transplantation of kidney organoids

Reaggregated kidney organoids at day 3 of *in vitro* culture were transplanted as previously described^8^. Because the mouse strains used as donor animals were maintained on a hybrid background, immunodeficient NOD/SCID mice were used as the host animals, and anaesthetised by peritoneal administration of normal saline containing 0.75 mg/kg medetomidine, 4.0 mg/kg midazolam, and 5.0 mg/kg butorphanol^36^. After surgery, atipamezole was administered as an anaesthetic antagonist. The grafted organoids were harvested at 7 days after transplantation (day 10).

### Flow cytometry analysis and sorting

Embryonic kidneys at E11.5 were dissociated as described above. After blocking in normal mouse serum (Thermo Fisher Scientific; 31881), staining was carried out for 30 min on ice in Hanks’ balanced saline solution (Thermo Fisher Scientific; 14185-052) containing 1% BSA and 0.035% NaHCO_3_. The following primary antibodies were used: anti-Itga8 (R&D Systems; BAF4076); anti-Pdgfra (Biolegend; 135907); anti-CD31 (BD Horizon; 562939); and anti-Flk-1 (BD Horizon; 562941). For Itga8 staining, an anti-goat secondary antibody conjugated with Alexa Fluor 488 (Thermo Fisher Scientific) or PE-streptavidin (BD Pharmingen; 554061) was used. Data were obtained using a FACS SORP Aria (BD Biosciences) and analysed with FlowJo software (TreeStar Inc.).

For quantification of CD31+ and tdTomato+ cells by flow cytometry analysis, the transplanted organoids were harvested at day 7 after transplantation. For dissociation, the samples were incubated with 2 mg/ml collagenase (Sigma; C9407) for 30 min, manually minced in PBS, and incubated for 45 min with DMEM/F12 medium containing 2.4 U/ml dispase (Thermo Fisher Scientific; 17105-041), 2 mg/ml collagenase, 50 µg/ml DNase (Roche, 11284932001), 2 mM CaCl_2_, and 10% FBS. The tissues were disrupted every 15 min by pipetting 10 times. Following the dissociation, the samples were treated with RBC lysis buffer (Thermo Fisher Scientific; 00-4333-57) for 10 min on ice to haemolyse the erythrocytes. The cells were stained for CD31 (BD Horizon; 562939) as described above. The percentages of tdTomato+ cells among CD31+ cells were examined in five organoids per group. The flow cytometric data were presented as mean ± s.e.m.

### Immunostaining of sections

Paraffin sections were subjected to antigen retrieval in citrate buffer. Next, paraffin or frozen sections were washed three times with PBS and blocked by incubation with 1% BSA in PBS for 1 h at room temperature. The sections were incubated overnight with primary antibodies at 4 °C, followed by incubation with secondary antibodies conjugated with Alexa Fluor 488, 568, 594, 633, or 647 for 90 min at room temperature. Nuclei were counterstained with 4,6-diamidino-2-phenylindole (Roche). The following primary antibodies were used: chick anti-GFP (Abcam; ab13970); rabbit anti-Cx40 (Alpha Diagnostic; CX40-A); mouse anti-E-cadherin (BD Biosciences; 610181); rabbit anti-CD31 (Abcam; ab28364); rat anti-CD31 (BD Biosciences; 557355); rabbit anti-Six2 (Proteintech; 11562-1-AP); rabbit anti-red fluorescent protein (RFP) (Rockland; 600-401-379); rat anti-TER-119 (MBL; D062-3); and guinea pig anti-nephrin (Progen Biotechnik; GP-N2). Fluorescence images were captured by a confocal microscope (TSC SP8; Leica).

For colouring of arteries and veins, paraffin blocks of E12.5 and E13.5 embryos were serially sectioned and stained with an anti-CD31 antibody (Abcam; ab28364) using a BlueMap Kit and a Discovery System (Roche). Each section was scanned with a slide scanner (SCN400; Leica), and images of the same regions were selected for alignment with an ImageJ plugin (Stackreg). CD31+ structures that were continuous with the aorta in each section were coloured in red, while those continuous with the large veins were coloured in blue using Photoshop software (Adobe Systems Inc.). The resulting images were reconstituted into three-dimensional images using Imaris software (Bitplane).

### Whole-mount immunostaining

Embryonic kidneys or organoids were fixed in 4% paraformaldehyde in PBS for 30–60 min at 4 °C, or in methanol/PBS series for 5 min each at room temperature followed by rehydration. The samples were washed twice with 0.1% Triton X-100 in PBS at room temperature, and blocked twice in PBS containing 1% BSA (Sigma Life Science; A4503), 1% Triton X-100, and 0.2% dry skim milk for 1 h at room temperature^37^. The tissues were incubated overnight in siliconised tubes with primary antibodies on a shaker at 4 °C, washed three times with 0.1% Triton X-100 in PBS for 15 min, and incubated with secondary antibodies conjugated with Alexa Fluor 488, 568, 594, 633, or 647 overnight on a shaker at 4 °C. After immunostaining, the samples were serially dehydrated with 50%, 70%, and 100% ethanol (pH 9.0), and cleared with ethyl cinnamate^38^. The following primary antibodies were used: rabbit anti-Cx40 (Alpha Diagnostic; CX40-A); rat anti-CD31 (BD Biosciences; 557355); mouse anti-Sall1 (Perseus Proteomics; PP-K9814-00); rat anti-CK8 (Developmental Studies Hybridoma Bank, University of Iowa; Troma-I); goat anti-NRP2 (R&D Systems; AF567); chick anti-GFP (Abcam; ab13970); mouse anti-E-cadherin (BD Biosciences; 610181); rabbit anti-Six2 (Proteintech; 11562-1-AP); and guinea pig anti-Nephrin (Progen Biotechnik; GP-N2). Three-dimensional fluorescence images were captured with a confocal microscope (TSC SP8; Leica) and reconstructed using Imaris (Bitplane) or LAS X (Leica) software.

### Quantification and statistical analysis

Student’s *t*-test was applied for statistical analysis of differences between two groups. Differences with values of p < 0.01 were considered statistically significant.

## Supplementary information

Supplemental info
