# Supplementary material for: Reconstitution of the embryonic kidney identifies a donor cell contribution to the renal vasculature upon transplantation
Source: Sci Rep. 2019 Feb 4;9:1172. doi: 10.1038/s41598-018-37793-z (PMC6362047; doi:10.1038/s41598-018-37793-z)

Supplemental information

Reconstitution of the embryonic kidney identifies a donor cell contribution to the renal vasculature upon transplantation

Yoichi Murakami[1], Hidekazu Naganuma[1,2], Shunsuke Tanigawa[1], Toshihiko Fujimori[3], Masatoshi Eto[2], and Ryuichi Nishinakamura[1]*

[1]Department of Kidney Development, Institute of Molecular Embryology and Genetics, Kumamoto University, Kumamoto 860-0811, Japan

[2]Department of Urology, Graduate School of Medical Sciences, Kyushu University, Fukuoka 812-8582, Japan

[3]Division of Embryology, National Institute for Basic Biology, Aichi 444-8787, Japan

***E-mail: ryuichi@kumamoto-u.ac.jp**

Supplementary Figure S1 Arterial and venous development in mouse embryos

(A, B) Examples of CD31-stained images of serially sectioned E12.5 (A) and E13.5 (B) embryos. ao: aorta; k: kidney; vc: vena cava; UB: ureteric bud.

(C, D) Arteries (red) and veins (blue) at E12.5 (C) and E13.5 (D) reconstructed from coloured serial sections. CD31+ vein-like cell clusters were detected. Arrows: renal arteries budding from the aorta (ao); arrowhead: collateral artery connecting the renal arteries and the common iliac artery.

(E, F) Whole-mount immunostaining of arteries (Cx40), veins (Nrp2), and UBs (CK8) in the E12.5 and E13.5 kidney.

(G, H) Magnified and digitised images of (E, F), respectively. Nrp2+ vein-like cell clusters were observed along the Cx40+ arteries.

Three mice were examined at each stage and representative images are shown. Scale bars: 100 μm.

Supplementary Figure S2 Vascular development in embryos and organoids

(A) Whole-mount staining of a freshly isolated E14.5 kidney from a wild-type mouse. Cx40+ arterioles (red) were detected in the spaces between the branching UBs (CK8: green). Scale bar: 100 μm.

(B, C) Section staining of kidney organoids with and without ECs after culture for 7 days *in vitro*. CD31+ cells existed in the interstitial space (arrows), especially in the organoids with ECs. Scale bar: 100 μm.

(D) Vascularization of kidney organoids with and without Tie2Cre;tdTomato+ ECs after culture for 7 days *in vitro*. The areas of tdTomato+ vasculature against those of the organoids were calculated as percentages. The tdTomato+ areas were calculated as the particles in split red channel images using Image J software (https://imagej.net/ImageJ). Representative images are shown in the right panels of Fig. 5B, D. *$p<0.05$ (n=3 per group).

Supplementary Figure S3 Vascular development upon transplantation

(A) Tie2Cre;tdTomato intact kidney (E11.5) at 7 days after transplantation. Scale bar: 200 μm.

(B) Flow cytometry analysis of CD31 and tdTomato in (A), showing the dominance of donor-derived cells over ECs in the transplanted organoids. The percentage of tdTomato+ donor cells among CD31+ ECs was 71.8 ± 1.05% (n =3).

(C) Sorting of Tie2Cre;tdTomato E11.5 dissociated kidney cells. CD31+ and/or Flk1+/tdTomato+ cells were sorted as ECs (left), and the cells in the CD31−/Flk1−/tdTomato− fraction (left) was further sorted into Itga8+ NP and Pdgfra+ SP fractions (right).

(D) Anuclear red blood cells in transplanted kidney organoids. The organoids generated with or without exogenous ECs (n=3 per group) were analysed at day 7 post-transplantation by anti-TER-119 immunostaining (red). Hoxb7-GFP UBs were detected by the anti-GFP antibody. Scale bars: 50 μm.

Supplementary Figure S1

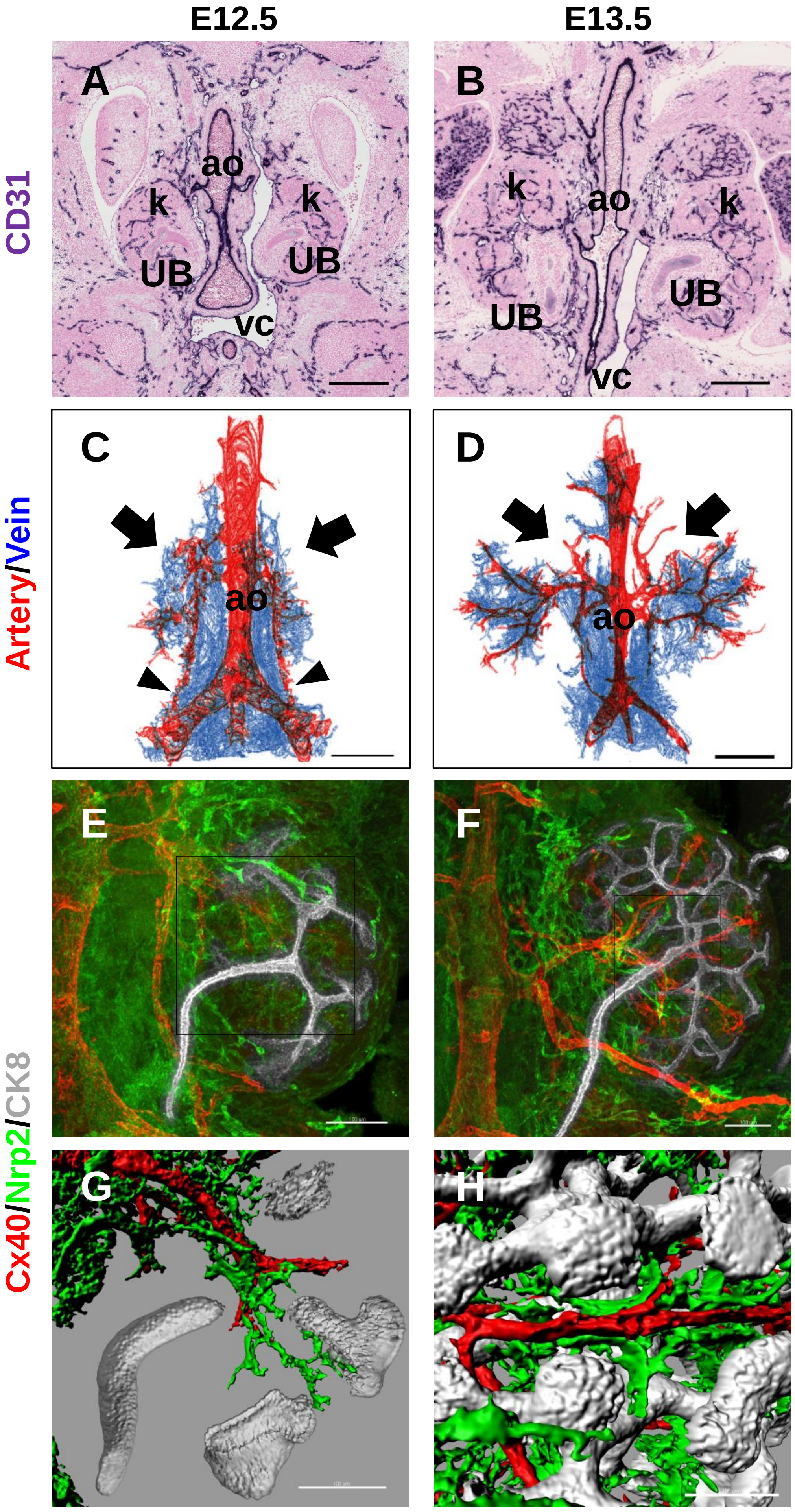

Supplementary Figure S2

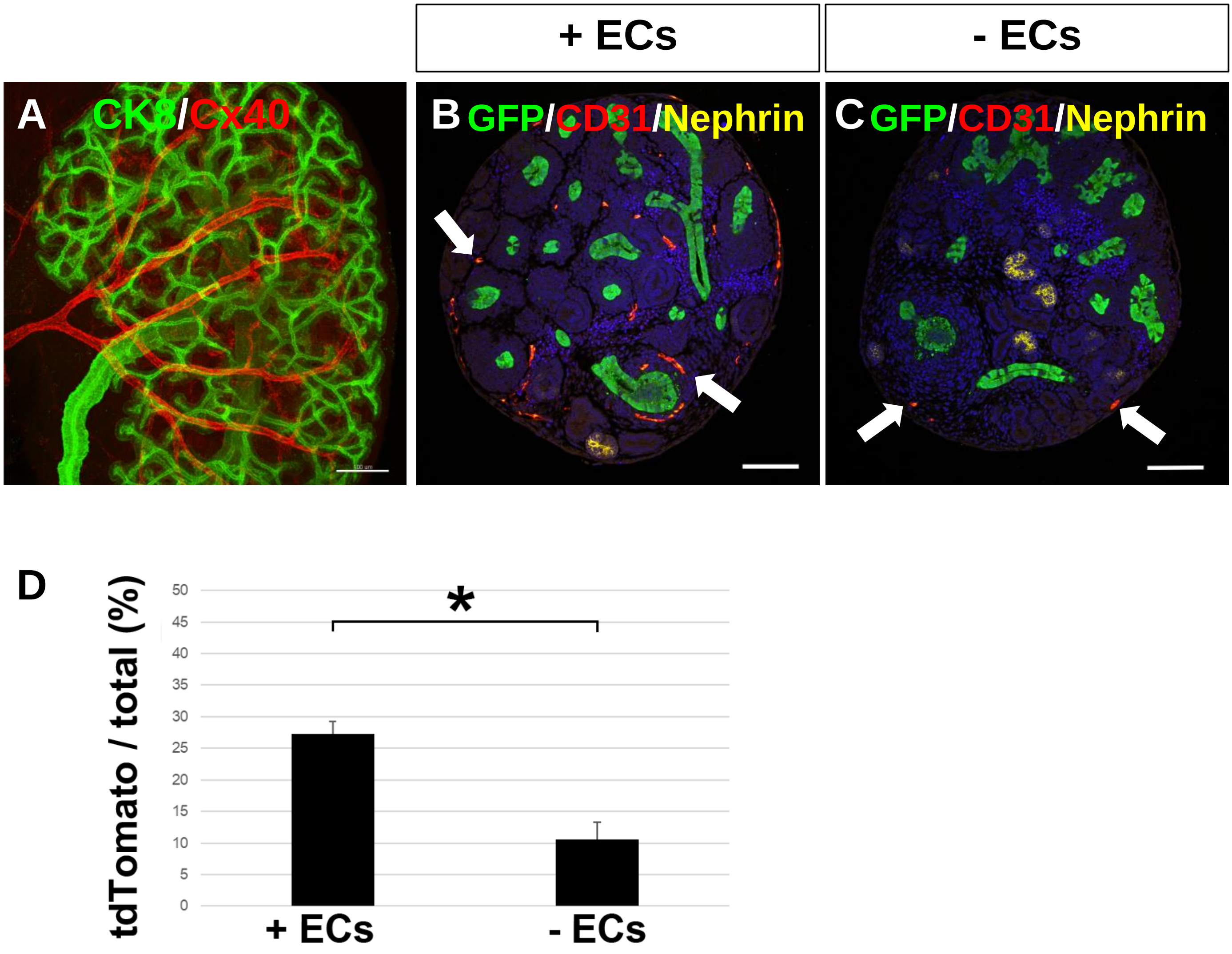

Supplementary Figure S3

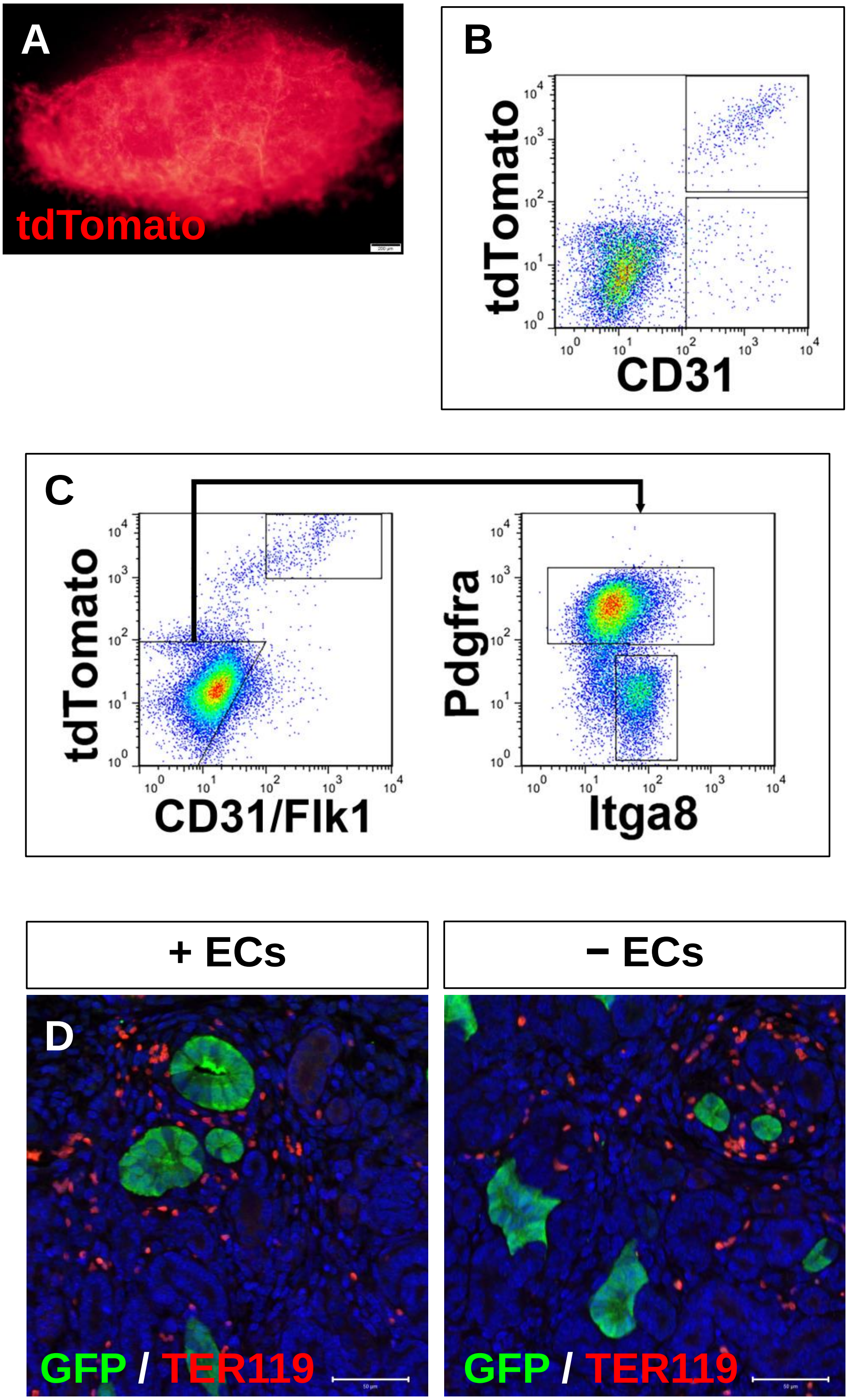

Supplement: Supplementary file 1 — Supplemental info [file 41598_2018_37793_MOESM1_ESM.pdf]
